# Use of supplemental oxygen therapy in idiopathic pulmonary fibrosis: an observational real-life study in 16 003 patients

**DOI:** 10.1136/bmjresp-2025-003153

**Published:** 2025-10-10

**Authors:** Claire Marant-Micallef, Manon Belhassen, Jean-Michel Fourrier, Maeva Nolin, Nadège Bornier, Stéphane Jouneau, Michael Kreuter, Katerina Samara, Vincent Cottin

**Affiliations:** 1 PELyon, Lyon, France; 2 Association Fibroses Pulmonaires France, Bordeaux, France; 3 Respiratory Medicine, Pontchaillou Hospital, Rennes, France; 4 Departments of Pneumology, Marienhaus Clinic Mainz, Mainz, Germany; 5 F Hoffmann-La Roche Ltd, Basel, Switzerland; 6 UMR 754, INRAE, Université Claude Bernard Lyon 1, F-69100, Villeurbanne, France; 7 Centre de Référence des Maladies Pulmonaires Rares, Service de Pneumologie, F-69000, Hospices Civils de Lyon, ERN-LUNG, Lyon, France

**Keywords:** Idiopathic Pulmonary Fibrosis, Long Term Oxygen Therapy (LTOT), Clinical Epidemiology

## Abstract

**Background and objectives:**

The use of long-term oxygen therapy (LTOT) in idiopathic pulmonary fibrosis (IPF) is poorly studied. We assessed the proportion of patients with IPF receiving LTOT and compared the risk of death according to LTOT exposure.

**Methods:**

Using the French national healthcare claims database, the use of LTOT and antifibrotics was studied in patients newly diagnosed with IPF from 1 January 2012 to 31 December 2019, followed until 31 December 2021. An adjusted Cox regression model was used to compare the risk of death by LTOT use, using exposure to antifibrotics and LTOT as time-dependent variables.

**Results:**

Among 16 003 patients newly diagnosed with IPF, 4559 (28.5%) initiated LTOT during follow-up: median time to initiation was 273 days and median duration was 336 days. The proportion of patients initiating LTOT was 23.2% among those not receiving antifibrotics (78.5% of study population) and 42.0% in those treated by antifibrotics at inclusion (7.7%), with respective median time to LTOT initiation of 110 and 590 days, and respective median LTOT duration of 308 and 294 days. Patients exposed to LTOT had a significantly higher risk of death compared with those who were not (HR: 2.9 (95% CI: 2.8 to 3.0) among those without antifibrotics; 2.1 (95% CI 1.9 to 2.3) among those with concomitant antifibrotics).

**Conclusions:**

The use of LTOT is limited among patients with IPF, even those receiving antifibrotics. The association between LTOT and mortality suggests that LTOT use is a marker of severity. Guidelines dissemination would help clinicians adopt appropriate LTOT management in patients with IPF and chronic respiratory failure.

**WHAT IS ALREADY KNOWN ON THIS TOPIC**

- Real-life data about the use of long-term oxygen therapy (LTOT) in patients with idiopathic pulmonary fibrosis (IPF) and corresponding patients’ characteristics are scarce.

**WHAT THIS STUDY ADDS**

- The study shows that 28.5% of the patients newly diagnosed with IPF initiated LTOT over their follow-up.
- LTOT was associated with higher risk of death compared with no exposure to LTOT or to antifibrotics, suggesting that LTOT is predominantly used at a late stage of the disease.

**HOW THIS STUDY MIGHT AFFECT RESEARCH, PRACTICE OR POLICY**

- The results suggest that the use of antifibrotics may delay the need for LTOT use, although patients receiving antifibrotics may have fewer comorbidities and may receive more comprehensive IPF management compared with non-treated patients.
- The use of LTOT may be considered as a proxy of disease severity and poor prognosis.

## Introduction

 Idiopathic pulmonary fibrosis (IPF) is a chronic progressive interstitial lung disease of unknown aetiology, causing a significant decline in lung function and worsening dyspnoea, leading to poor quality of life.^1^ When untreated, the median survival time from diagnosis is 2–4 years. The disease mainly occurs in older adults, with an incidence estimated at 2.8–18 cases per 100 000 people in Europe and North America.^2^

Antifibrotic treatments available since 2012 (pirfenidone) and 2015 (nintedanib) decrease lung function decline in patients with IPF^3 4^ and improve overall survival,^5 6^ although they do not stop disease progression. Both drugs are recommended for the treatment of IPF following a multidisciplinary discussion.^7^ In France, antifibrotics are reimbursed to treat patients with forced vital capacity ≥50% and diffusion capacity for carbon monoxide (DLCO) ≥30%; however, a significant proportion of patients remain untreated,^8^ while guidelines for the use of antifibrotics in patients with IPF were published in France in 2013 and updated in 2017 and 2023.^9^,^12^ National and international guidelines recommend the use of supplemental, long-term oxygen therapy (LTOT) in patients with chronic respiratory failure including IPF at rest and/or on exertion to improve exercise capacity and health-related quality of life.^7 11 13 14^ Specifically, French criteria to initiate LTOT in IPF patients are the following: PaO2≤55 mm Hg (7.3 kPa) measured at rest in a stable state on two occasions; or PaO2 between 56 and 60 mm Hg (7.3–8.0 kPa) in the presence of at least one of the following criteria: polycythaemia (haematocrit >55%), signs of pulmonary hypertension, documented signs of right heart failure, non-apnoeic nocturnal desaturations.^13 15^ However, studies highlight some practical and social barriers to its use.^16 17^ To date, data regarding the real-life use of LTOT in patients with IPF, and its association with survival, are sparse.^18 19^

The objective of the study was to assess the real-life use of LTOT in patients with IPF. Specifically, the aims were to describe the proportion of patients who received LTOT, their sociodemographic and clinical characteristics, as well as the time to initiation and the duration of LTOT, according to whether they were receiving an antifibrotic treatment or not. In an exploratory analysis, the risk of death was also compared between periods during which patients were using LTOT to periods during which they were not using LTOT over the course of the disease, accounting for antifibrotic treatment use.

## Methods

### Data source

This retrospective, population-based, cohort study was based on the French National Health System claims *database (Système National des Données de Santé, SNDS).* It contains anonymous and exhaustive individual information on sociodemographic characteristics, non-hospital reimbursed healthcare expenditures (without corresponding medical diagnoses), hospital discharge summaries (International Classification of Diseases (ICD) 10-code-based), and death, for people living in France (68 million inhabitants). The SNDS does not provide direct information on behavioural or clinical baseline characteristics (tobacco smoking, body mass index, pulmonary function test results, etc), laboratory or tests results, drug dispensation during a hospital stay, or cause of death. This claims database currently covers more than 98% of the population of France.^20^

### Study population and periods

Incident patients with IPF were identified through a first hospitalisation with a main or related diagnosis of IPF (ICD-10 code: J84.1), a first reimbursement of pirfenidone or nintedanib, or at least one reimbursement linked to a long-term disease status, that is, full coverage for a condition requiring long-term care and particularly costly treatment associated with a diagnosis of IPF, occurring between 1 January 2012 and 31 December 2019, that is, the inclusion period. Patients also had to be covered by the national health insurance general scheme between 2007 and 2021, to ensure exhaustive analytical data. We excluded patients aged less than 50 years, or with at least one differential diagnosis including pneumoconiosis, connective tissue diseases or sarcoidosis as the main, related or associated diagnosis of hospitalisation or as long-term disease status identified over the 5 years prestudy period preceding the inclusion date. The date of the first identification of one of the three inclusion criteria was defined as the inclusion date. Patients with lung transplantation or LTOT prior to the inclusion date were excluded. Patients were followed from the inclusion date to the end of the study period, that is, 31 December 2021, or to the last patient’s health record (defined as the last care recorded before a 6-month period without any reimbursed care), lung transplantation or death.

We described patients overall, those not treated with antifibrotics at any time during follow-up, and those treated with antifibrotics at the time of inclusion. We described and compared characteristics of patients initiating LTOT to those of patients not initiating LTOT using χ^2^ and Wilcoxon tests. Next, to assess the association between LTOT use and survival, we defined four subgroups of patients: patients initiating LTOT over the follow-up (with or without antifibrotics initiation at any time), and patients not initiating LTOT over the follow-up (with or without antifibrotics initiation at any time).

### Variables

Sociodemographic characteristics included age at inclusion, gender and free-access-to-care status identified in the 12 months preceding the inclusion date, as a proxy of social deprivation. Clinical characteristics were the mode of IPF detection (ie, hospitalisation with an IPF diagnosis, long-term disease status for IPF, or dispensation of an antifibrotic treatment during the inclusion period, whichever occurred first), comorbid conditions identified in the 12 months before the inclusion date, Charlson Comorbidity Index^21^ and death.

Patients were considered exposed to LTOT if no discontinuation in dispensing LTOT devices occurred for 3 months or more over the whole follow-up period. The time to LTOT initiation was defined as the number of days between the inclusion date and the first dispensing of supplemental oxygen therapy considered as LTOT. The duration of LTOT was defined as the number of days from the first LTOT dispensing to the end of the follow-up. The time to antifibrotic initiation was defined as the number of days between the inclusion date and the first dispensing of antifibrotic treatment during the follow-up. The survival time was defined as the time from the inclusion date to the date of death.

The following confounding factors were included as covariates in the comparative analyses: age, gender and proxies of IPF severity identified in the 12 months before inclusion date, that is, number of acute respiratory-related hospitalisations, number of visits to lung specialists, Charlson Comorbidity Index, presence of comorbidities such as pulmonary hypertension, malnutrition and chronic obstructive pulmonary disease (COPD) or emphysema.

### Statistical analysis

Descriptive statistics were used to describe sociodemographic and clinical characteristics, time to LTOT initiation and LTOT duration, overall and by subgroup of patients. We used the Kaplan-Meier method to describe time from inclusion to death according to LTOT initiation and antifibrotic treatment (yes/no). Quantitative variables were described with the sample size, mean, SD, Q1–Q3, minimum and maximum, whereas categorical variables were described using sample size of each modality and relative percentages. Cox proportional hazard models adjusted on confounding factors were used to compare the risk of death in patients when they were exposed to LTOT with/without antifibrotics, using the periods when patients did not receive LTOT or antifibrotics as a reference, and considering exposure to antifibrotics and to LTOT as time-dependent variables. Confounding factors included the following: age at inclusion, gender, Charlson Comorbidity Index and proxies of IPF severity, that is, the number of respiratory-related hospitalisations, of visits to office-based lung specialists or hospital physicians, presence of COPD or emphysema, of malnutrition or of pulmonary hypertension identified through long-term disease status or a diagnosis related to a hospitalisation.

### Patient and public involvement

A patients’ representative has been involved at each step of the study, that is, from protocol writing to publication. More specifically, his role was crucial to ensure relevancy of outcomes measured and in the interpretation of the results.

## Results

### Study population

**Figure 1:**
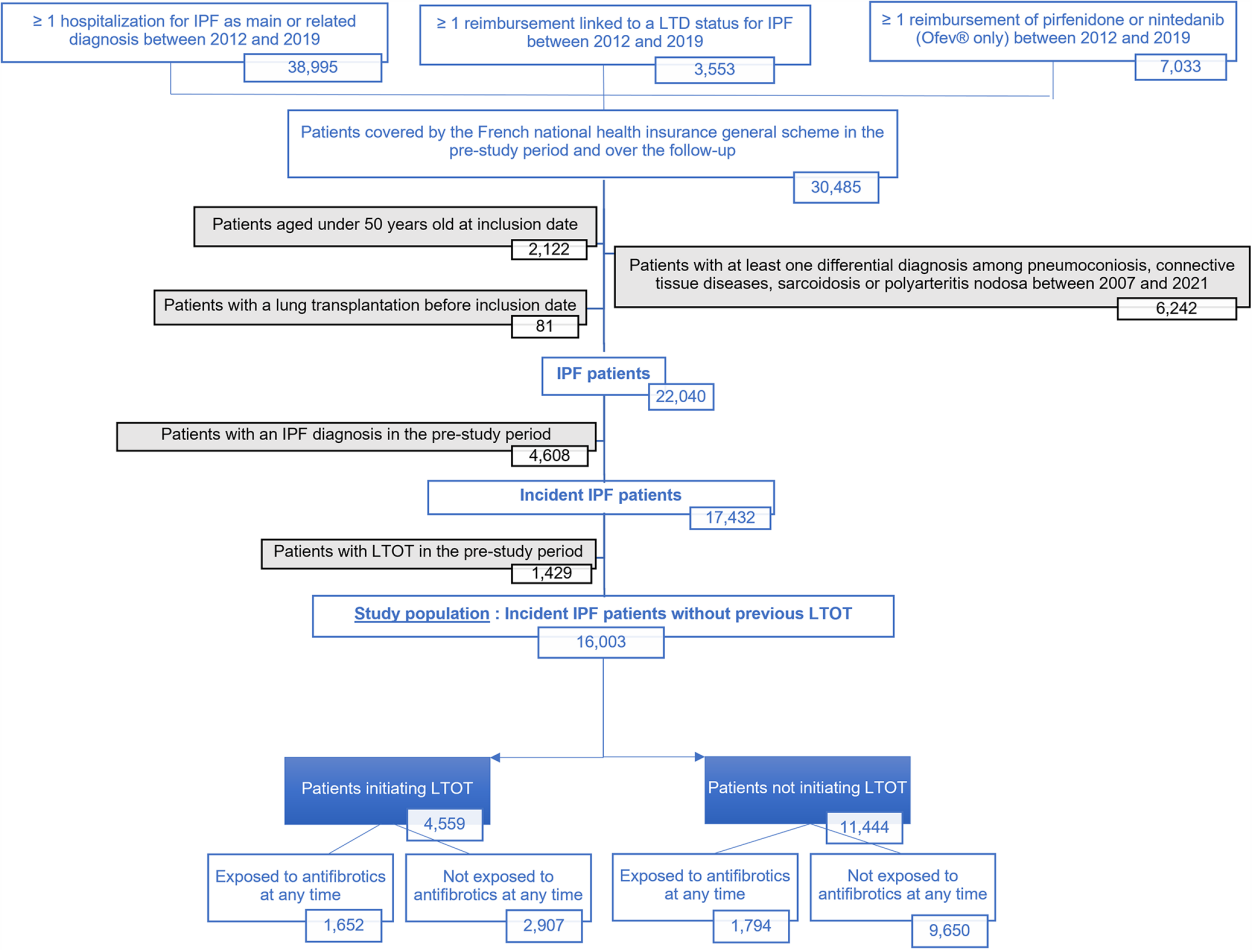
Selection of the incident IPF patients with no previous LTOT before inclusion and subgroups of the study population. IPF, idiopathic pulmonary fibrosis; LTOT, long-term oxygen therapy.

We identified 16 003 patients newly diagnosed with IPF during the study period, who had not received LTOT at baseline (figure 1). Their mean follow-up duration was 3.0 years (SD: 2.4): more than half of the patients (n=9195, 57.5%) died over the follow-up, 5.0% were lost to follow-up and 0.9% had lung transplantation. The mean age of the study population was 74.7 years±10.5 SD. The mean Charlson Comorbidity Index was 4.5±2.5, reflecting a very comorbid population, including 20.0% of patients with COPD and/or emphysema; 1.6% patients were classified with concomitant pulmonary hypertension (table 1).

**Table 1:** Characteristics of the study population according to antifibrotic treatment

	Entire study population N=16 003	Patients not treated by AF N=12 557	Patients treated by AF at inclusion date N=1228
Males, n (%)	9836 (61.5%)	7149 (56.9%)	987 (80.4%)
Mean age at inclusion date (in years) (SD)	74.7 (10.5)	75.4 (10.9)	73.5 (8.2)
Full coverage, n (%)	602 (3.8%)	485 (3.9%)	50 (4.1%)
Type of first IPF detection, n (%)			
Hospitalisation for IPF	13 677 (85.5%)	11 985 (95.4%)	≤10
Reimbursement linked to a long-term disease status for IPF	1113 (7.0%)	572 (4.6%)	≤10
Reimbursement of pirfenidone or nintedanib	1213 (7.6%)	0 (0%)	1213 (98.8%)
Death during the follow-up, n (%)	9195 (57.5%)	7498 (59.7%)	604 (49.2%)
Comorbidities			
Mean Charlson Comorbidity index (SD)	4.5 (2.5)	4.7 (2.6)	3.9 (1.8)
Depression and anxiety	6958 (43.5%)	5757 (45.8%)	410 (33.4%)
Hypertensive conditions	3617 (22.6%)	3172 (25.3%)	166 (13.5%)
Diabetes mellitus	3264 (20.4%)	2580 (20.5%)	274 (22.3%)
Chronic obstructive pulmonary disease and/or emphysema	3196 (20.0%)	2172 (17.3%)	557 (45.4%)
Ischaemic heart disease	2993 (18.7%)	2330 (18.6%)	273 (22.2%)
Other forms of heart disease	2933 (18.3%)	2583 (20.6%)	141 (11.5%)
Malnutrition	2703 (16.9%)	2495 (19.9%)	88 (7.2%)
Heart failure	1591 (9.9%)	1477 (11.8%)	43 (3.5%)
Disorders of lipoprotein metabolism and other dyslipidaemias	1010 (6.3%)	841 (6.7%)	64 (5.2%)
Lung cancer	544 (3.4%)	470 (3.7%)	39 (3.2%)
Sleep apnoeas	491 (3.1%)	379 (3.0%)	15 (3.0%)
Pulmonary hypertension	263 (1.6%)	238 (1.9%)	≤10
Pulmonary embolism	221 (1.4%)	206 (1.6%)	≤10

AF, antifibrotic therapy; IPF, idiopathic pulmonary fibrosis.

### Use of antifibrotics in the study population

More than three-quarters of the population (n=12 557, 78.5%) never received antifibrotics over the whole follow-up; 1228 patients (7.7%) were treated with antifibrotics at inclusion, and antifibrotics were initiated during follow-up in 13.9% of patients. Table 1 presents the sociodemographic and clinical characteristics of the study population according to the use of antifibrotics. Patients not treated with antifibrotics were 56.9% males. They had a mean age of 75.4 years. Their death rate was 59.7% over a mean follow-up of 2.9 years. They had a Charlson mean score of 4.7, with 45.8% of patients with anxiety or depression. Conversely, patients treated with antifibrotics from inclusion date were 80.4% males, with a mean age of 73.5 years. Their death rate was 49.2% over a mean follow-up of 3.2 years. Their Charlson mean score was 3.9, with 45.5% of patients with COPD and/or emphysema as a comorbidity. Similar data about the 2218 patients who initiated antifibrotics over the follow-up are presented in the online supplemental table S1.

### Use of LTOT in the study population

Overall, 4559 patients (28.5%) initiated LTOT over the follow-up, with a median time to initiation of 273.0 days, and a median duration of LTOT of 336.0 days. Table 2 presents sociodemographic and clinical characteristics separately in patients initiating LTOT and patients not initiating LTOT. The mean age at inclusion of patients initiating LTOT (75.4 years) was significantly higher than the age of patients not initiating LTOT (74.4 years), and there were significantly more males in patients who initiated LTOT (65.7% vs 59.8%, respectively). The Charlson score at index date was similar between the two groups of patients (4.4 in patients initiating LTOT vs 4.6). The proportion of patients with COPD/emphysema (27.3%) and with ischaemic heart diseases (21.0%) was significantly higher in patients initiating LTOT than in patients not initiating LTOT (respectively, 17.0% and 17.8%). However, the proportion of patients with hypertensive conditions (20.8%) or malnutrition (13.8%) was significantly lower in patients initiating LTOT than in patients not initiating LTOT (respectively, 23.3% and 18.1%).

**Table 2:** Patients’ characteristics according to LTOT initiation

	Patients without LTOT throughout follow-up (N=11 444)	Patients with initiation of LTOT during the follow-up (N=4559)	P value*
Males, n (%)	6843 (59.8%)	2993 (65.7%)	<0.0001
Mean age at inclusion date in years (SD)	74.4 (10.8)	75.4 (9.6)	<0.0001
Full coverage	456 (4.0%)	146 (3.2%)	0.0176
Death during the follow-up, n (%)	5737 (50.1%)	3458 (75.8%)	<0.0001
Mean Charlson Comorbidity Index (SD)	4.6 (2.6)	4.4 (2.2)	0.8177
Depression and anxiety	5040 (44.0%)	1918 (42.1%)	0.0233
Hypertensive conditions	2668 (23.3%)	949 (20.8%)	0.0007
Diabetes mellitus	2281 (19.9%)	983 (21.6%)	0.0209
Chronic obstructive pulmonary disease and/or emphysema	1951 (17.0%)	1245 (27.3%)	<0.0001
Ischaemic heart disease	2036 (17.8%)	957 (21.0%)	<0.0001
Other forms of heart disease	2173 (19.0%)	760 (16.7%)	0.0006
Malnutrition	2075 (18.1%)	628 (13.8%)	<0.0001
Heart failure	1165 (10.2%)	426 (9.3%)	0.1107
Disorders of lipoprotein metabolism and other dyslipidaemias	738 (6.4%)	272 (6.0%)	0.2572
Lung cancer	427 (3.7%)	117 (2.6%)	0.0002
Sleep apnoeas	371 (3.2%)	120 (2.6%)	0.0435
Pulmonary hypertension	173 (1.5%)	90 (2.0%)	0.0378
Pulmonary embolism	179 (1.6%)	42 (0.9%)	0.0017

* χ2 or Wilcoxon test.

LTOT, long-term oxygen therapy.

Among patients who did not receive antifibrotics, who represented the majority (78.5%) of the study population, 23.2% initiated LTOT, with a median time to initiation of 110 days (3.6 months, Q1–Q3: 18.0–598.0) and a median duration of LTOT until death or end of follow-up of 308 days (10.1 months, Q1–Q3: 98.0–679.0) (table 3). Among patients treated with antifibrotics at inclusion, 42.0% initiated LTOT, with a median time to initiation of 590 days (19.4 months, Q1–Q3: 242.5–1093.0) and a median duration of 294 days (9.7 months, Q1–Q3: 140.0–612.5).

**Table 3:** Time from inclusion to initiation and duration of LTOT by antifibrotic treatment status

	Overall N=16 003	Not treated by antifibrotics over the follow-up N=12 557	Treated by antifibrotics at inclusion N=1228
Time to LTOT initiation (in days)			
N (%)	4559 (28.5%)	2907 (23.2%)	516 (42.0%)
Mean (SD)	546.8 (652.6)	410.7 (599.4)	742.1 (614.2)
Median (Q1–Q3)	273.0 (36.0–867.0)	110.0 (18.0–598.0)	590.0 (242.5–1093.0)
Duration of LTOT dispensation (in days)			
Mean (SD)	487.7 (487.8)	468.4 (498.0)	432.0 (402.4)
Median (Q1–Q3)	336.0 (126.0–707.0)	308.0 (98.0–679.0)	294.0 (140.0–612.5)
Mean duration of follow-up (in years) (SD)	3.0 (2.4)	2.9 (2.5)	3.2 (1.7)

LTOT, long-term oxygen therapy.

### Mortality according to the use of LTOT and exposure to antifibrotics

Among the 9195 patients who died over the follow-up, 37.6% were receiving LTOT in the 3 months preceding death. Among patients who initiated LTOT (n=4559), 3458 (75.8%) died over the follow-up: among them, 2363 (68.3%) were not treated by antifibrotics, and 1095 (31.7%) received antifibrotics at any time (either at inclusion or later). The median time from LTOT initiation to death was 9.2 months (Q1–Q3: 3.3–19.2) in non-treated patients and 13.0 months (Q1–Q3: 6.0–23.5) in patients treated with antifibrotics.

**Figure 2:**
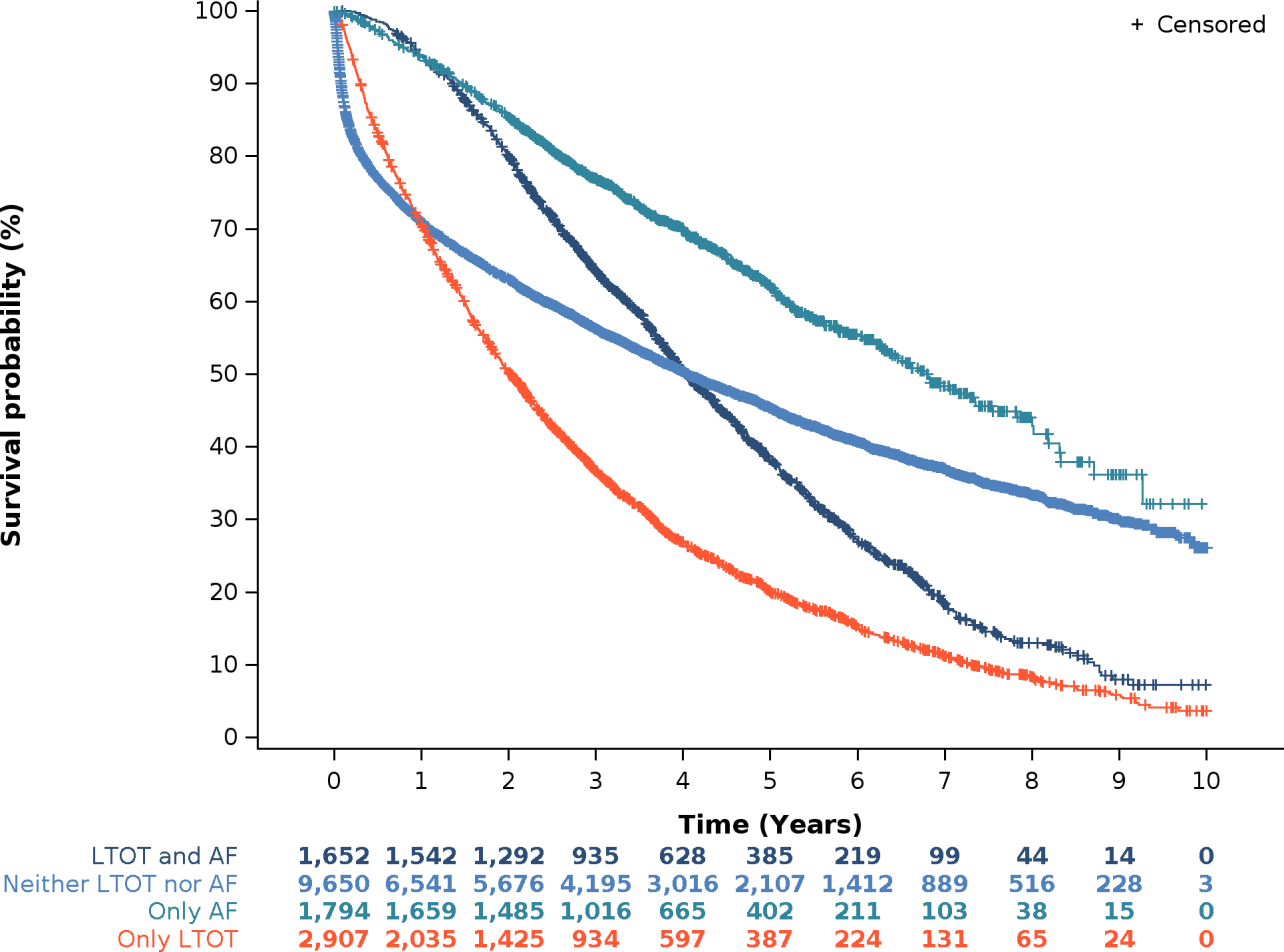
Survival time from inclusion according to exposure to LTOT and AF over the follow-up. AF, antifibrotics; LTOT, long-term oxygen therapy.

Overall, 9650 patients never received LTOT or antifibrotics over the follow-up, 1652 received both LTOT and antifibrotics at one point; 2907 had periods during which they received LTOT only, and 1794 had periods during which they received antifibrotics only, at any time over the follow-up (figure 1). The survival time from inclusion differed according to the exposure to antifibrotics and/or LTOT over the follow-up (figure 2): the median survival time was 9.7 months (Q1–Q3: 1.5–31.7) in patients who did not receive LTOT or antifibrotics, 18.3 months (Q1–Q3: 7.5–35.0) in those who used LTOT but not antifibrotics, 27.4 months (Q1–Q3: 15.7–44.2) in patients who used antifibrotics but no LTOT, and 34.7 months (Q1–Q3: 21.7–53.1) in those who received both LTOT and antifibrotics. Compared with not being exposed to LTOT nor to antifibrotics and adjusting for confounding factors, the probability of death was higher (HR: 2.10, IC_95%_: 1.92 to 2.31) while being exposed to both LTOT and antifibrotics, higher (HR=2.90, IC_95%_: 2.77 to 3.04) while being exposed to LTOT only, and lower (HR: 0.28, IC_95%_: 0.24 to 0.32) while being exposed to antifibrotics and not to LTOT.

## Discussion

Our study provides novel meaningful information about the dispensing of LTOT in patients newly diagnosed with IPF, also in association with antifibrotic treatment.

Several studies,^22^,^24^ including the present one, have shown that a small proportion of patients with IPF get treated with antifibrotics: here, patients with IPF who received an antifibrotic represented only 7.5% of the study population at inclusion, and 21.5% throughout the study period. This is in line with a similar German study based on claims data in which 11.6% of the newly diagnosed IPF patients were treated with antifibrotics at 5 years after diagnosis.^19^ We found that the proportion of newly diagnosed patients who initiated LTOT was also relatively low throughout the study period, that is, less than one-third (28.5%), contrasting with the high frequency of nocturnal hypoxaemia in IPF^25 26^ and the negative prognostic role of resting hypoxaemia in this condition.^27^ This proportion varied according to the antifibrotic treatment status: patients who were already treated by antifibrotics at inclusion or who initiated an antifibrotic in the year following inclusion (online supplemental table S2 in the electronic supplement) had higher rates of LTOT initiation (42.0% and 43.8%, respectively), whereas only 23.2% of the patients who never received an antifibrotic initiated LTOT at some point during the disease course. LTOT was initiated earlier in patients not receiving antifibrotics. We hypothesise that this could reflect a more severe disease in patients who are not treated with antifibrotics, thus requiring early LTOT, and the decision to only initiate supportive care including LTOT in patients with more severe disease, which is supported by the high mortality rate in this subgroup (59.7%) and a Charlson score of 4.7 (compared with 3.9 in treated patients). The data are also consistent with our previous studies showing that patients not receiving antifibrotics had greater all-cause mortality (cumulative all-cause mortality at 3 years of 50%) than antifibrotic-treated patients (cumulative all-cause mortality at 3 years of 26%–31% depending on the treatment).^8 22^ It is important to note that among a large group of patients who died during follow-up, 62.4% never received LTOT even during the 3 months preceding death, suggesting that the use of LTOT is low, even at an advanced stage of the disease.

The unadjusted survival time from inclusion of patients never exposed to LTOT or antifibrotics was shorter than that of patients exposed to either LTOT and/or antifibrotics. However, when adjusting for available confounding factors, we found that when patients were exposed to LTOT or both to LTOT and antifibrotics, they had a higher risk of death compared with when they were not exposed to LTOT or to antifibrotics. It should be noted that these results were based on observational data and were not adjusted for the severity of IPF as a confounding factor, because lung function and blood gas results are not available in the SNDS database. Although no conclusion of causality can be made, we hypothesise that the greater mortality observed in patients receiving LTOT generally reflects greater disease severity. The use of LTOT may be considered as a proxy of disease severity and poor prognosis, which heretofore has probably been underestimated. The median time from initiation of LTOT to death was 318 days in this study, compared with a median survival from the start of oxygen therapy (ambulatory or LTOT) of 537±74 days in a recent study.^28^ Although treated patients may be different from non-treated patients (eg, less comorbidities) and managed more comprehensively, our results further suggest that antifibrotic treatment may delay the need to initiate LTOT, which is highly meaningful for patients. Conversely, we found improved outcomes in patients who start antifibrotic treatment once LTOT has been initiated, consistent with a previous study showing an improved survival in patients with antifibrotics compared with untreated patients, regardless of LTOT.^22^

The limited proportion of patients initiating LTOT may be explained at least in part by the absence of international guidelines specific to IPF. International guidelines on oxygen use in chronic lung disease,^29 30^ and French IPF guidelines^11^ published after the study period now address indications for LTOT. Nevertheless, our findings highlight the need for appropriate testing for hypoxaemia to guide appropriate management decisions. From a patient’s perspective, LTOT may be difficult to accept, because of the burden of the device in everyday life and psychological and societal considerations. Further study is needed to assess the various patient-related, societal-related and physician-related barriers that hamper initiation of LTOT in patients with IPF,^31^,^33^ analogous to barriers to antifibrotic treatment.^34^

One of the strengths of the study is that we used a database almost exhaustive of the French population. It can thus be assumed that virtually all patients newly diagnosed with IPF in France between 2012 and 2019 were included, as our inclusion criteria included a comprehensive combination of claims frequently used by IPF patients (LTD status, IPF-related hospitalisations and specific drug reimbursement), even if we cannot exclude that a minority of patients was missed. Moreover, the data contained in the SNDS are highly reliable, as they are homogeneously coded and are not exposed to any memory bias, for instance. This provides a highly robust and detailed description of the patients’ population and their outcomes.

The main limitation of this study is the absence in the database of lung function variables or blood gas analyses, which prevented adjusting the comparative survival analysis on the most meaningful disease severity markers. However, LTOT may be used as a marker of IPF progression in future studies, when clinical disease severity indicators are not available. Due to the observational design, no causal relationship can be established regarding the observed association between antifibrotic use, LTOT and mortality. As we used claims data, detailed information about compliance to prescribed LTOT was not available for this study, although prescribers of LTOT in France do receive feedback about the individual compliance to LTOT. Also, we ignore the context of the IPF diagnosis (eg, expert centre, only based on CT), as it was only based on ICD-10 codes. Finally, the use of ambulatory oxygen therapy could not be assessed in this study.

In conclusion, in addition to providing a detailed description of patients’ characteristics and survival of IPF patients receiving LTOT and/or AF treatment, these results show that LTOT is used in a minority of patients with IPF, even at a late stage of disease. Although no clinical data were available to assess theoretical indications for LTOT, we speculate that LTOT might be underused in IPF, similar to antifibrotic therapy. These findings further underline a substantial need for prospective studies to better understand whether LTOT is appropriately offered to patients with IPF, for the dissemination of guidelines and education of clinicians to LTOT, and/or for informing patients about the expected benefits of LTOT when indicated.

## Supplementary material

10.1136/bmjresp-2025-003153 online supplemental file 1

## Data Availability

Data sharing not applicable as no datasets generated and/or analysed for this study.
