# Supplementary material for: Use of supplemental oxygen therapy in idiopathic pulmonary fibrosis: an observational real-life study in 16 003 patients
Source: BMJ Open Respir Res. 2025 Oct 10;12(1):e003153. doi: 10.1136/bmjresp-2025-003153 (PMC12516998; doi:10.1136/bmjresp-2025-003153)
Supplement: online supplemental file 1 [file bmjresp-12-1-s001.docx]

**Supplementary material**

**Use of supplemental oxygen therapy in idiopathic pulmonary fibrosis: an observational real-life study in 16,003 patients**

**Table S1.** Sociodemographic and clinical characteristics of the study population

|  | Entire study population  N=16,003 | | Patients not treated by AF  N=12,557 | | Patients treated by AF at inclusion date    N=1,228 | | Treated by AF ≤1 year from inclusion without LTOT before AF initiation  N=1,364 | | Treated by AF ≤1 year from inclusion with LTOT before AF initiation    N=166 | | Treated by AF >1 year from inclusion without LTOT before AF initiation  N=494 | | Treated by AF >1 year from inclusion with LTOT before AF initiation  N=194 | |
| --- | --- | --- | --- | --- | --- | --- | --- | --- | --- | --- | --- | --- | --- | --- |
| Male, n (%) | 9,836 (61.5%) | | | 7,149 (56.9%) | | 987 (80.4%) | | 1,071 (78.5%) | | 124 (74.7%) | 371 (75.1%) | 134 (69.1%) | |  |
| Mean age at inclusion date (in years) (SD) | 74.7 (10.5) | | | 75.4 (10.9) | | 73.5 (8.2) | | 72.0 (8.4) | | 74.2 (8.4) | 70.4 (8.0) | 70.7 (8.4) | |  |
| Free-access-to-care status, n (%) | 602 (3.8%) | | | 485 (3.9%) | | 50 (4.1%) | | 41 (3.0%) | | ≤ 10 | 17 (3.4%) | ≤ 10 | |  |
| Type of first IPF detection, n (%) | | | |  | |  | |  | |  |  |  | |  |
| Hospitalization for IPF | 13,677 (85.5%) | | | 11,985 (95.4%) | | ≤ 10 | | 957 (70.2%) | | 144 (86.7%) | 417 (84.4%) | 167 (86.1%) | |  |
| Reimbursement linked to a LTD status for IPF | 1,113 (7.0%) | | | 572 (4.6%) | | ≤ 10 | | 407 (29.8%) | | 22 (13.3%) | 77 (15.6%) | 27 (13.9%) | |  |
| Reimbursement of pirfenidone or nintedanib | 1,213 (7.6%) | | | 0 (0%) | | 1,213 (98.8%) | | 0 (0%) | | 0 (0%) | 0 (0%) | 0 (0%) | |  |
| Death during the follow-up, n (%) | 9,195 (57.5%) | | | 7,498 (59.7%) | | 604 (49.2%) | | 659 (48.3%) | | 133 (80.1%) | 189 (38.3%) | 112 (57.7%) | |  |
| **Comorbidities** |  | | |  | |  | |  | |  |  |  | |  |
| Mean Charlson Comorbidity index (SD) | 4.5 (2.5) | | | 4.7 (2.6) | | 3.9 (1.8) | | 3.5 (1.5) | | 4.1 (1.9) | 3.4 (1.6) | 3.6 (1.5) | |  |
| **Patients' comorbidities, n (%)** | |  | |  | |  | |  | |  |  |  | |  |
| Depression and anxiety | 6,958 (43.5%) | | | 5,757 (45.8%) | | 410 (33.4%) | | 496 (36.4%) | | 48 (28.9%) | 185 (37.4%) | 62 (32.0%) | |  |
| Hypertensive conditions | 3,617 (22.6%) | | | 3,172 (25.3%) | | 166 (13.5%) | | 177 (13.0%) | | 23 (13.9%) | 50 (10.1%) | 29 (14.9%) | |  |
| Diabetes Mellitus | 3,264 (20.4%) | | | 2,580 (20.5%) | | 274 (22.3%) | | 254 (18.6%) | | 37 (22.3%) | 79 (16.0%) | 40 (20.6%) | |  |
| Chronic obstructive pulmonary disease and/or emphysema | 3,196 (20.0%) | | | 2,172 (17.3%) | | 557 (45.4%) | | 308 (22.6%) | | 44 (26.5%) | 73 (14.8%) | 42 (21.6%) | |  |
| Ischemic heart disease | 2,993 (18.7%) | | | 2,330 (18.6%) | | 273 (22.2%) | | 232 (17.0%) | | 45 (27.1%) | 77 (15.6%) | 36 (18.6%) | |  |
| Other forms of heart disease | 2,933 (18.3%) | | | 2,583 (20.6%) | | 141 (11.5%) | | 129 (9.5%) | | 21 (12.7%) | 45 (9.1%) | 14 (7.2%) | |  |
| Malnutrition | 2,703 (16.9%) | | | 2,495 (19.9%) | | 88 (7.2%) | | 76 (5.6%) | | 12 (7.2%) | 18 (3.6%) | 14 (7.2%) | |  |
| Heart failure | 1,591 (9.9%) | | | 1,477 (11.8%) | | 43 (3.5%) | | 33 (2.4%) | | 16 (9.6%) | 14 (2.8%) | ≤ 10 | |  |
| Disorders of lipoprotein metabolism and other dyslipidemias | 1,010 (6.3%) | | | 841 (6.7%) | | 64 (5.2%) | | 65 (4.8%) | | ≤ 10 | 16 (3.2%) | 14 (7.2%) | |  |
| Lung cancer | 544 (3.4%) | | | 470 (3.7%) | | 39 (3.2%) | | 24 (1.8%) | | ≤ 10 | ≤ 10 | 0 (0%) | |  |
| Sleep apneas | 491 (3.1%) | | | 379 (3.0%) | | 41 (3.3%) | | 52 (3.8%) | | ≤ 10 | 15 (3.0%) | ≤ 10 | |  |
| Pulmonary hypertension | 263 (1.6%) | | | 238 (1.9%) | | 17 (1.4%) | | ≤ 10 | | 0 (0%) | ≤ 10 | ≤ 10 | |  |
| Pulmonary embolism | 221 (1.4%) | | | 206 (1.6%) | | ≤ 10 | | ≤ 10 | | 0 (0%) | ≤ 10 | 0 (0%) | |  |

**Table S2**: Time from inclusion to initiation and duration of long-term oxygen therapy by subgroup of patients

|  | **Overall**  **N=16,003** | **Not treated by AF over the follow-up**  **N = 12,557** | **Treated by AF at inclusion**  **N = 1,228** | **Treated by AF ≤1 year from inclusion, without LTOT before AF initiation**  **N = 1,364** | **Treated by AF ≤1 year from inclusion with LTOT before AF initiation**    **N=166** | **Treated by AF >1 year from inclusion, without LTOT before AF initiation**  **N = 494** | **Treated by AF >1 year from inclusion with LTOT before AF initiation**  **N=194** |
| --- | --- | --- | --- | --- | --- | --- | --- |
| **Time to LTOT initiation (in days)** |  |  |  |  |  |  |  |
| N (%) | 4,559 (28.5%) | 2,907 (23.2%) | 516 (42.0%) | 597 (43.8%) | 166 (100.0%) | 179 (36.2%) | 194 (100.0%) |
| Mean (SD) | 546.8 (652.6) | 410.7 (599.4) | 742.1 (614.2) | 846.0 (590.9) | 57.7 (72.6) | 1,521.9 (700.7) | 666.4 (589.5) |
| Median (Q1 – Q3) | 273.0 (36.0 – 867.0) | 110.0 (18.0 - 598.0) | 590.0 (242.5 - 1,093.0) | 715.0 (385.0 - 1,196.0) | 18.5 (9.0 - 82.0) | 1,375.0 (973.0 - 2,059.0) | 505.5 (231.0 - 1,013.0) |
|  |  |  |  |  |  |  |  |
| **Duration of LTOT dispensation (in days)** |  |  |  |  |  |  |  |
| Mean (SD) | 487.7 (487.8) | 468.4 (498.0) | 432.0 (402.4) | 471.7 (427.3) | 746.0 (576.4) | 427.0 (374.6) | 808.9 (545.3) |
| Median (Q1 - Q3) | 336.0 (126.0 – 707.0) | 308.0 (98.0 - 679.0) | 294.0 (140.0 - 612.5) | 343.0 (154.0 - 679.0) | 574.0 (329.0 - 980.0) | 329.0 (133.0 - 616.0) | 700.0 (413.0 - 1,085.0) |
|  |  |  |  |  |  |  |  |
| Mean duration of follow-up (in years) (SD) | 3.0 (2.4) | 2.9 (2.5) | 3.2 (1.7) | 3.6 (1.8) | 2.3 (1.6) | 5.2 (2.0) | 4.1 (1.8) |
